# A Promising Candidate in Tendon Healing Events—PDGF-BB

**DOI:** 10.3390/biom12101518

**Published:** 2022-10-20

**Authors:** Yixuan Chen, Li Jiang, Kexin Lyu, Jingwei Lu, Longhai Long, Xiaoqiang Wang, Tianzhu Liu, Sen Li

**Affiliations:** 1 School of Physical Education, Southwest Medical University, Luzhou 646000, China; 2 Spinal Surgery Department, The Affiliated Traditional Chinese Medicine Hospital of Southwest Medical University, Luzhou 646000, China; 3 Neurology Department, The Affiliated Traditional Chinese Medicine Hospital of Southwest Medical University, Luzhou 646000, China

**Keywords:** platelet-derived growth factor-BB, tendon injury, tendinopathy, tendon repair, mechanism

## Abstract

Tendon injuries are one of the most common musculoskeletal disorders for which patients seek medical aid, reducing not only the quality of life of the patient but also imposing a significant economic burden on society. The administration of growth factors at the wound site is a feasible solution for enhancing tendon healing. Platelet-derived growth factor-BB (PDGF-BB) has a well-defined safety profile compared to other growth factors and has been approved by the Food and Drug Administration (FDA). The purpose of this review is to summarize the role of PDGF-BB in tendon healing through a comprehensive review of the published literature. Experimental studies suggest that PDGF-BB has a positive effect on tendon healing by enhancing inflammatory responses, speeding up angiogenesis, stimulating tendon cell proliferation, increasing collagen synthesis and increasing the biomechanics of the repaired tendon. PDGF-BB is regarded as a promising candidate in tendon healing. However, in order to realize its full potential, we still need to carefully consider and study key issues such as dose and application time in the future, so as to explore further applications of PDGF-BB in the tendon healing process.

## 1. Introduction

Musculoskeletal disorders are characterized by high morbidity and disability in the epidemiology of many countries and are therefore an important branch of public health [1]. Tendon-related musculoskeletal disorders are relatively common in clinical practice and are marked by regressive changes in the mechanical properties and cellular structure of tendons [2]. Tendons consist of parallel aligned longitudinal collagen fibers and dense connective tissue that connect muscle and bone in the musculoskeletal system. Tendons are required to withstand the forces generated by muscle loading in order to transmit muscle forces to the bones. Overuse of the tendon is thought to be a major factor in the development of tendinopathy [3]. Tendinopathy can occur in all age groups and is a common condition that affects the quality of life and tendon function of patients. Due to the poor vascular network and low metabolic rate of tendons, tendon healing, which includes the three overlapping phases of inflammation, proliferation, and remodeling, is a lengthy process.

Some in vitro studies have shown that different growth factors including fibroblast growth factor-2 (FGF-2), transforming growth factor-β (TGF-β), vascular endothelial growth factor (VEGF) and platelet-derived growth factor (PDGF) may play a key role in regulating angiogenesis, matrix formation and cell proliferation in tendons, with the goal of improving tendon healing [2]. However, it was found that exogenous delivery of FGF-2 in a canine model did not improve the biomechanical or molecular properties of the treated tendons [4]. Furthermore, some studies have shown that the local delivery of TGFβ can improve the histological and biomechanical properties of tendons, but being a growth factor associated with scar and adhesion formation, TGFβ may lead to abnormal scar formation [5]. Moreover, because the tendon is hypovascular, some vascularization of it can provide a better healing result. However, in this regard, VEGF has detrimental effects on tendon healing as it leads to a high level of hypoxia-inducible factor 1 (HIF-1)/VEGF-induced and matrix metallopeptidase 3 (MMP-3)-supported angiogenesis, which ultimately produces the result of poorer recovery of the tendon’s biomechanical properties [6]. As a pleiotropic growth factor, PDGF plays a key role in physiological processes such as tissue healing. In particular, recombinant human PDGF can be used for clinically targeted tissue repair as well as regeneration due to its natural and potent wound healing stimulating activity [7]. As a universal ligand subtype of PDGF tyrosine kinase receptor, PDGF-BB is known for its mitogenic, chemotactic and angiogenic activities, and has been suggested as a promising candidate for tendon repair [8]. PDGF-BB has a stronger chemotaxis than FGF-2, and the locally delivered PDGF-BB can improve the biomechanical properties of the repaired tendon by recruiting tendon cells from adjacent tendon tissues to the site of injury, thereby promoting the healing of the regenerated tendon [9,10]. Differing from TGFβ, PDGF-BB can reduce scar formation rather than forming abnormal scarring. α-smooth muscle actin (α-SMA) is a central marker of fibrotic tissue and fibrosis in general. Meier et al. showed that PDGF-BB can reduce α-SMA^+^ cells in Achilles tendon healing. They concluded that the reduction in the amount of α-SMA protein in the presence of PDGF-BB could be judged as a positive, anti-fibrotic outcome, which was thought to result in less scar formation during the healing process [11,12,13]. Moreover, in the aspect of tendon vascularization, the application of PDGF-BB is the opposite of that of VEGF, the former having been shown to be beneficial. In addition, PDGF-BB has been approved by the FDA and is used in clinical settings for other applications. These clinical approvals and biological properties make it a safer and more promising growth factor [14]. On the other hand, Meier et al. reported the results of mature tendon cells possessing a higher proportion of dense area in the presence of PDGF-BB-treated tendons. They found that such clusters of mature tendon cells have not been reported before. From a maturation perspective, this tendon cell cluster demonstrates accelerated healing [13]. This is an interesting finding that prompts the desire for further attention to be paid to PDGF-BB.

PDGF-BB, as the most studied heterodimer in the PDGF growth factor family, is the only dimer that can bind to three different surface platelet-derived growth factor receptors (PDGFRs) and trigger different signal pathways. Its role in tendon healing is multifaceted. Once PDGF-BB combines with three different surface PDGFRs, it will initiate the PDGF signal cascade, in which different cellular processes are affected through different signal pathways [11,15,16].

Several studies have demonstrated the role of PDGF-BB in promoting tendon healing. Nevertheless, the elaborate mechanisms have not been fully elucidated. Hence, the purpose of this review is to summarize the role and mechanism of PDGF-BB in tendon healing, to further explore the methods of PDGF-BB in promoting tendon healing in the future and to provide a theoretical basis for follow-up researchers.

**Figure 1:**
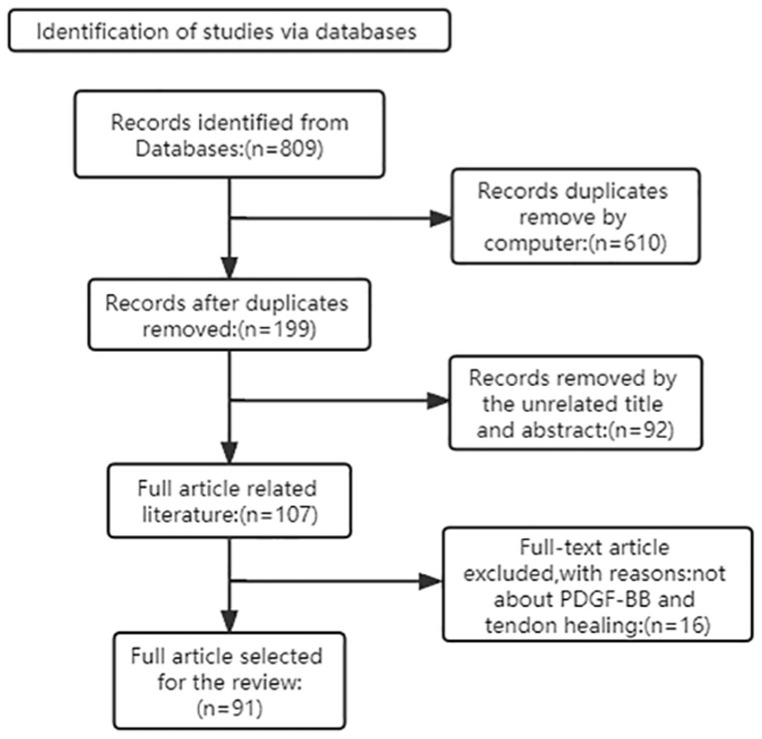
Article Retrieval Flow Chart with inclusion and exclusion process.

Search strategy. (i) Search site: Articles are from PubMed, a database of papers on biomedical science. (ii) Database: MEDLINE. (iii) Keywords: platelet-derived growth factor-BB, PDGF-BB, tendon injury, tendinopathy, tendon healing. (iv) Boolean algorithm: (“platelet-derived growth factor-BB” OR “PDGF-BB”) AND (“Tendinopathy” OR “Tendon injury” OR “Tendon healing”). (v) Retrieval timeframe: We searched the selected journals published from 1982 to 2022. (vi) Inclusion and exclusion criteria: Articles were included if the topic was related to platelet-derived growth factor-BB and tendon healing, and the article type was a review or an experimental paper. The search process is shown in Figure 1.

## 2. PDGF-BB in Tendon Healing: Experimental Studies

A number of experimental studies have shown that PDGF-BB has an important role in the process of tendon healing [9,11,13].

Yoshikawa, Thomopoulos and Haupt et al. all investigated the benefits of PDGF-BB in tendon healing through in vitro experiments. Yoshikawa et al. investigated the stimulatory effects of PDGF-BB on cell proliferation and matrix synthesis in rabbit tendons. They found that PDGF-BB stimulated matrix synthesis and cell proliferation in rabbit tendons in a dose-dependent manner in vitro, supporting the possibility of the clinical application of PDGF-BB for enhancing intrinsic tendon metabolism [17]. Thomopoulos et al. cultured fibroblasts isolated from dog flexor tendons in a medium and then added PDGF-BB, which was found to cause a 13-fold and 2-fold increase in cell proliferation and collagen synthesis, respectively, compared to the control [18]. Haupt et al. used recombinant human platelet-derived growth factor-BB (rhPDGF-BB) to study grafted, cultured equine superficial finger flexor tendons (SDFT), and type I collagen expression was markedly higher than in untreated control specimens; in contrast, type III collagen gene expression was suppressed. This indicates a positive matrix healing and tendon remodeling effect of rhPDGF-BB [19].

On the other hand, many in vivo experiments have demonstrated the important role of PDGF-BB in tendon healing. Hildebrand et al. investigated the effects of PDGF-BB application on mature male New Zealand White rabbits and then found that the application of PDGF-BB to the ruptured right medial collateral ligament dramatically improved the failure absorption energy, ultimate load and ultimate elongation values of the femoral–MCL–tibial complex compared to the no growth factor group. Furthermore, higher doses of PDGF-BB were able to better refine the structural properties of the femoral–MCL–tibial complex compared to lower doses of PDGF-BB. Simultaneously, the PDGF-BB alone treatment group produced better results compared with a combination of PDGF-BB plus TGF-β1. In this study, the ability of PDGF-BB to improve healing in the early stages of ligament repair has been confirmed [20].

Furthermore, Kovacevic et al. set up a rat rotator cuff repair model and randomly divided the rats into five groups: the repair-only group, collagen scaffold-only group and three collagen scaffold groups with different doses of PDGF-BB. The results of the experiment showed that after 5 days, delivery of rhPDGF-BB on the scaffold showed a dose-dependent response in terms of cell angiogenesis and proliferation compared to the repair-only and scaffold-only groups. This result also confirms that the delivery of rhPDGF-BB on collagen scaffolds strengthens angiogenesis and cell proliferation in the early stages of healing. At the same time, this study illustrates the importance of ongoing understanding and optimization of growth factor delivery systems that can lead to an enhanced healing environment to improve structural integrity and reduce re-injury rates after tendon healing [8].

Unlike others, Younesi et al. applied PDGF-BB growth factor solution to heparinized electrochemically aligned collagen sutures (ELAS), allowed the solution to be completely absorbed by the otherwise dry sutures and then used it to suture the completely torn right third toe flexor muscle of mature White Leghorn chickens. The results showed that the combination of heparinized ELAS with PDGF-BB improved biomechanics and vascularity during tendon healing 12 weeks after primary repair [21].

Ultimately, Evrova et al. established a rabbit Achilles tendon complete tear model and then used electrospun tubes made of DegraPol^®^ (DP) (polyester urethane), conveying PDGF-BB, as an implant to improve tendon healing. In vivo evaluation after three weeks showed positive results, with the delivery of PDGF-BB via bioactive DP tubing leading to a 2-fold increase in the tensile strength on treated tendons, with no additional pro-fibrotic effects, thus providing a prospective bioactive implant for clinical applications in the field of tendon healing [9].

In conclusion, these studies suggest that PDGF-BB has a positive effect on tendon healing by promoting angiogenesis, cell proliferation and collagen production as well as increasing the biomechanics of the repaired tendon. The biological responses embodied by PDGF-BB in different animal experiments are shown in Table 1.

## 3. The Basis of PDGF-BB Efforts: PDGFRs

PDGF-BB exerts its cellular action by binding to the receptor and entering the cell. LIANG et al. mentioned that increasing PDGF-BB concentration was ineffective in facilitating cell proliferation when the number of its receptors was limited and they were all occupied [26]. PDGF-BB, the only universal heterodimer in the PDGF family that binds to three different PDGFRs (PDGFRαα, PDGFRαβ and PDGFRββ), initiates a signaling cascade upon binding to its receptor [11]. In different cells, PDGF-BB can elicit different downstream cascade signaling pathways, which include phosphoinositide 3-kinase (PI3K), phospholipase C-γ (PLCγ), mitogen-activated protein kinase (MAPK) and Janus kinase (JAK)/STAT, among others, and they are involved in different cellular and developmental processes. These are discussed in more detail in the article by Heldin, Andrae et al. [29,30].

**Figure 2:**
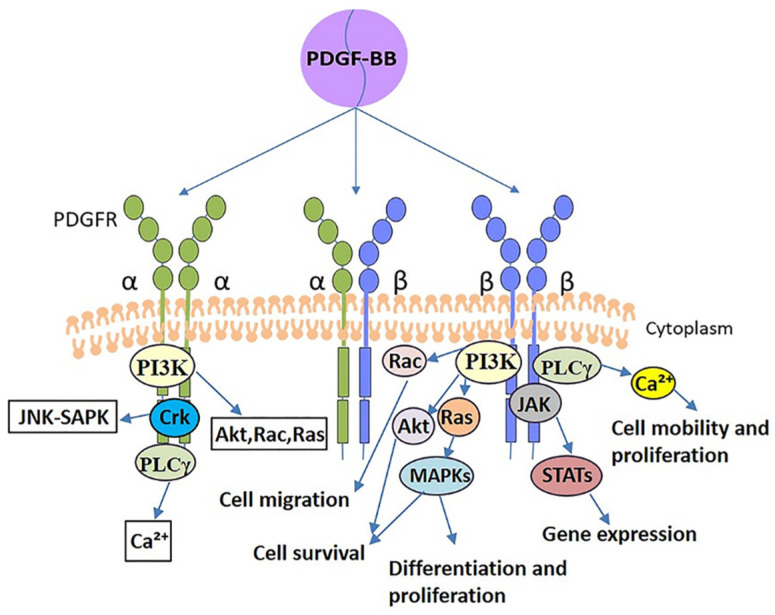
A simplified representation of the signaling action after PDGF-BB binding to PDGFRβ, which is mainly involved in tendon cell-related activities, has been made, and other mechanistic processes have been omitted. Green circles and blue circles are receptors for PDGF; α, α receptor of PDGF; β, β receptor of PDGF.

Although the heterodimeric receptor complexes of PDGF may have distinctive properties, most of the information on the function and role of homodimers is accessible. Both α and β receptor homodimers transmit very effective mitogenic signals, but their effects on the actin filament system are distinct. β receptors can mediate the formation of circular actin structures on the dorsal surface of cells, whereas α receptors cannot [31]. Furthermore, the activation of α receptors inhibits chemotaxis of certain cells, including fibroblasts, etc. Inversely, the activation of β receptors stimulates chemotaxis [32]. Since both α and β receptors differ in their binding to PDGF isoforms and transduction of signals, the cellular response to PDGF stimulation will depend on the type of PDGFRs expressed by the cells. Fibroblasts were reported to express both α and β receptors in the research of Heldin et al., but β receptors are expressed at higher levels [30]. Tendon cells are specialized fibroblasts, and therefore β receptors may have an even more important role in the cellular processes of tendon cells, as demonstrated in the study by Rolf et al. that showed the higher proliferative capacity of tendon cells is partly due to their higher levels of PDGFR β expression [33]. In a nutshell, PDGFRs are the basis for the efforts of PDGF-BB. The signal transductions after PDGF-BB binding to PDGFRs are shown in Figure 2. A simplified representation of the signaling action after PDGF-BB binding to PDGFRβ, which is mainly involved in tendon cell-related activities, has been made, and other mechanistic processes have been omitted.

## 4. The Mechanisms of PDGF-BB in Tendon Healing

Among the events that promote tendon healing, the administration of growth factors at the wound site is a viable strategy. After tendon injury, PDGF-BB is secreted by platelets along with other growth factors. When PDGF-BB is applied as a potent chemokine, it can accelerate tendon healing by promoting mitosis and angiogenesis. As a universal heterodimer of PDGF, PDGF-BB initiates a signaling cascade once it combines with its receptor, and different signaling pathways affect different cellular processes [11]. It should be noted that the time point of application and dose have a significant impact on its effectiveness. Since the endogenous release of PDGF-BB is during the inflammatory and early proliferative phase, it should be administered within the first two weeks after injury [34].

**Figure 3:**
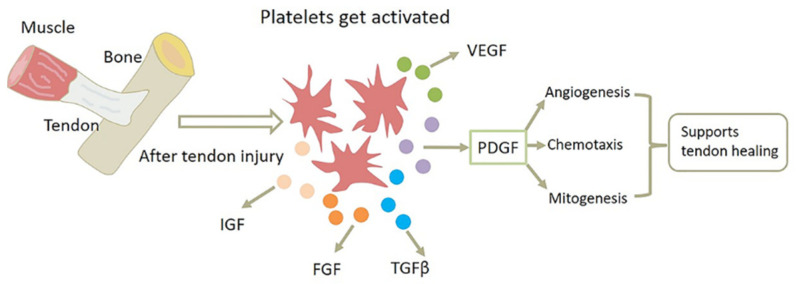
PDGF promotes angiogenesis and uses its chemotactic and mitogenic properties to support tendon healing.

**Figure 4:**
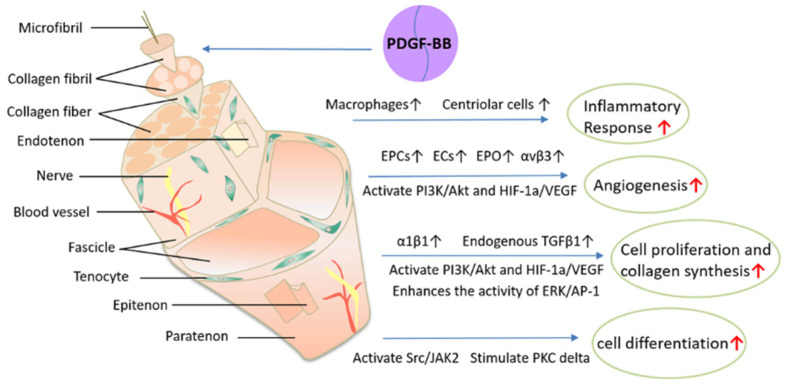
The mechanisms of PDGF-BB in tendinopathy. Black↑, increased; red↑, promoted.

Different molecular elements and mechanisms are present in the different phases of tendon healing. The inflammatory phase tends to occur within the first hour after tendon injury; immediately after injury, platelets and inflammatory cells (such as macrophages and neutrophils) release a mixture of cytokines and growth factors that are attracted to the wound site and produce tumor necrosis factor (TNF) or growth factors involved in neovascularization, such as PDGF, fibroblast growth factor (FGF), VEGF and so on, as shown in Figure 3. Of them, PDGF is one of the most important angiogenic factors regulating angiogenesis, and it can promote angiogenesis by upregulating related growth factors and other means. Additionally, PDGF can support tendon healing through its chemotactic and mitogenic properties. The high chemotactic activity of PDGF suggests that it may also be involved in attracting inflammatory cells to sites of platelet release during the inflammatory phase, thereby killing pathogens and performing an active effect. Notably, epidermal growth factor, a mitogenic factor, has no impact on monocyte migration, suggesting that not all mitogenic proteins are chemotactic for inflammatory cells [35]. In the process of cell proliferation of tendon healing, fibroblasts recruited from the tendon sheath and tendon proliferate as a result of numerous growth factors produced at the wound site, while gradually migrating into the wound, and then extracellular matrix protein (ECM) components, such as collagen, begin to be synthesized. During this tissue-forming phase, PDGF-BB not only promotes cell proliferation and collagen synthesis, but also influences cell migration through its powerful chemotactic effects. It helps to reduce cell density at the wound site, preparing for the remodeling phase [11]. Ultimately, during the remodeling phase of tendon healing, the tissue usually gradually changes from fibrous to scar-like tendon tissue. Compared to uninjured tendons, the development of scarred tendon tissue may result in poorer mechanical, structural and biological properties. Nevertheless, PDGF-BB can positively impact collagen deposition and cross-linking, improving the biomechanical properties of the healing tendon and promoting regenerative tendon healing rather than fibrotic healing [9,11]. The mechanisms of PDGF-BB in the treatment of tendinopathy are shown in Figure 4.

### 4.1. PDGF-BB Enhances Inflammatory Response

The inflammatory phase, as the first stage of tendon healing, is an important driver for normal tissue healing [36]. While chronic and uncontrolled inflammation is harmful, acute and controlled inflammation has a positive protective effect on the tissues [37]. A previous study reported that transient recruitment and activation of macrophages and fibroblasts by PDGF-BB in the inflammatory response can significantly accelerate the normal tissue repair process [38].

Successful tissue healing requires an initial inflammatory response [39]. A previous study demonstrated that recombinant platelet-derived growth factor-BB (rPDGF-BB) enhances the acute inflammatory response in the early stages of repair and also recruits and activates wound macrophages [40]. Macrophages are potentially important for effective debridement and the subsequent healing of injured tendons [36]. It has two phenotypes, with M1 macrophages exerting a pro-inflammatory effect and M2 macrophages contributing to tissue healing and remodeling [41]. M1 macrophages establish the initial immune environment, which together with neutrophils perform pathogen killing to re-establish barrier protection and tissue clearance to remove dead tissue to facilitate cell recruitment and ECM deposition. M2 macrophages then release growth factors (TGFβ1, IGF1, VEGF, PDGF and so on) to stimulate tissue repair during the later stages of inflammatory tendon healing [39]. In addition, Zhang et al. showed that PDGF-BB can inhibit interleukin-1β (IL-1β)-induced inflammation [42]. It provides a new perspective on the use of PDGF-BB in inflammation.

The series of inflammatory responses to harmful stimuli is considered an essential physiological process in the body [37]. However, persistent inflammation is thought to lead to fibrosis, which is an undesirable consequence of tendon healing [36]. Consequently, the balance of the inflammatory program is very important for tendon healing [39].

### 4.2. PDGF-BB Speeds Up Angiogenesis

Due to the characteristics of connective tissue, tendons have a weak vascular network, which makes tendon healing a long-term process; therefore, the angiogenic response is a key event in the tendon healing process. This is a complex multistep process [43]. Studies have shown that PDGF-BB can induce an angiogenic response during tendon healing, thereby promoting increased nutrient and metabolic waste elimination and further accelerating tendon healing [11].

The PI3K signaling pathway is one of the major signaling pathways induced by the activation of PDGF-BB and contributes to many cellular processes, including angiogenesis as well as cell proliferation, survival and motility [12,44]. Interaction between PDGF-BB and PDGFR-β can activate PI3K [45]. The activated PI3K is able to turn the cell membrane lipid 4,5-bisphosphatidylinositol (PI(4,5)P2) into 3,4,5-trisphosphatidylinositol (PI(3,4,5)P3), while forming 3,4-bisphosphatidylinositol (PI(3, 4)-P2) [46]. The binding of signaling proteins with pleckstrin homology (PH) structural domains to PI(3,4,5)P3 can facilitate the phosphorylation of Akt by phoinositide-dependent kinase 1 (PDK1). PI(4,5)P2 and PI(3, 4)-P2 are also capable of binding to the PH domain of Akt as the second messengers. Such binding induces a conformational shift in Akt, exposing two amino acid residues, namely serine 473 and threonine 308, which are, respectively, phosphorylated by PDK1 and phoinositide-dependent kinase 2 (PDK2). Phosphorylation of both amino acids is required for the activation of Akt, which induces downstream signaling molecules resulting in diverse cellular functions [47]. PI3K/Akt has been shown to be a key signaling pathway involved in hypoxia-inducible factor 1α (HIF-1α) activation [48], and VEGF happens to be one of the most important genes regulated by HIF-1α [47]. Although the dose of VEGF determines whether it induces normal angiogenesis, co-expression of PDGF-BB allows normalization of abnormal angiogenesis caused by high doses of VEGF [49]. On the other hand, the PI3K/Akt signaling pathway can promote the proliferation and migration of endothelial progenitor cells (EPCs) because of the overexpression of PDGFR-β, so as to foster the process of angiogenesis [12]. Indeed, EPCs, as specific bone marrow-derived progenitor cells, are capable of differentiating into endothelial cells (ECs), in addition to their role as themselves for angiogenesis and endothelial monolayer regeneration [12]. ECs’ proliferation is necessary for the formation of new blood vessels [50]. As components of capillaries, ECs along with pericytes carry all the genetic information used to form tubes, branches and the entire capillary network [51].

PDGF-BB, as a growth factor known to enhance mitogenesis, can promote angiogenesis by directly regulating the proliferation of vascular endothelial cells and inducing cell migration via PDGFR-β [50,52]. Generally, PDGF receptors are not expressed in cultures of macrovascular endothelial cells, whereas PDGF receptors have been reported to be expressed in microcapillary endothelial cells in various places such as rat brains or wounds [53,54,55]. In angiogenesis, microvascular endothelial cells respond to angiogenesis by increasing proliferation, the expression of protein hydrolases and the synthesis of specific extracellular matrix components. These are the processes that are essential for ECs to form new endothelial vessels and, together with pericytes, to remodel new capillaries. Battegay et al. proved that PDGF-BB can facilitate angiogenesis by binding with PDGFR-β receptors to directly induce the proliferation and migration in a specific phenotype of ECs [50].

Apart from that, PDGF-BB has also been proved to stimulate angiogenesis by inducing erythropoietin (EPO) production in stromal cells [56]. Experiments by Uslu et al. showed that EPO has a positive effect on biological responses during tendon healing, including angiogenesis [57]. With the aim of identifying the signaling pathways involved in the regulation of EPO expression by PDGF-BB, Xue et al. performed Affymetrix gene array analysis in their study and found that Klf5 (encoding transcription factor Klf5) and Atf3 (encoding transcription factor Atf3) were strongly upregulated by PDGF-BB. They made further measurements of the expression of these two genes by quantitative real-time PCR (qRT-PCR), and compared to buffer-treated stromal cells, Klf5 increased only 8-fold, while Atf3 increased 70-fold under PDGF-BB activation. After using specific siRNA probes, they found that Atf3 mediated the transcriptional upregulation of PDGF-BB-stimulated EPO expression, whereas treatment with Klf5 siRNA did not influence the promoter activation of EPO. As the sequence analysis of the EPO promoter domain did not expose Atf3 shared binding sites, it is likely that Atf3 may not work on the EPO promoter directly. In the final analysis, they found that at the molecular level, the PDGF-BB-PDGFR-β signaling system can trigger the EPO promoter by inducing the Atf3 transcription factor related to Sp1 and c-Jun [56]. Based on previously published findings, EPO may contribute to angiogenesis in the following mechanisms: Above all, by stimulating the proliferation, differentiation and mobilization of EPCs [58,59,60]; via direct induction of EC proliferation and migration [61,62,63,64]; and through the increase in vascular stability to further support angiogenesis [65].

Eventually, angiogenesis mediated by the regulation of specific integrins by PDGF-BB may be an important parameter for successful tendon repair [22]. Angiogenesis hinges on the adhesion interactions of vascular cells. During the healing process, the basement membrane zone of blood vessels expresses some adhesion proteins including fibrin, fibronectin and von Willebrand factor, and the αvβ3 integrin is the endothelial cell receptor for these adhesion proteins. The αvβ3 integrin can induce angiogenesis by triggering a calcium-dependent signaling pathway that causes ECs migration. It has been identified as a marker of angiogenic vascular tissue and plays a key role in angiogenesis induced by various stimuli [66]. Harwood et al. previously reported the stimulatory effect of PDGF-BB on αvβ3 expression in vitro. They found that PDGF-BB increases αvβ3 integrin expression during flexor tendon repair, and subsequently confirmed by semi-quantitative reverse transcription polymerase chain reaction (RT-PCR) that PDGF-BB increases αv mRNA expression in flexor cells within the tendon by up to 3-fold. Therefore, they suggest that PDGF-BB may enhance the success of tendon repair by regulating specific integrin-mediated angiogenesis [22]. Taken together, PDGF-BB accelerates the formation of new blood vessels and thus speeds up the rate of tendon healing.

### 4.3. PDGF-BB Stimulates Tendon Cell Proliferation and Increases Collagen Synthesis

Tendon cells are specialized fibroblasts. PDGF-BB has been demonstrated to induce the mitosis and proliferation of fibroblasts. Several experimental studies have shown that PDGF-BB can stimulate the proliferation of tendon cells and, similarly, has a positive stimulatory effect on collagen synthesis in tendon cells.

Above all, it has been shown that PDGF-BB can be involved in fibroblast proliferation, transformation and collagen synthesis by activating the PI3K/AKT signaling pathway [67]. Previous studies have found that the activation of the PI3K/AKT signaling pathway can enhance the proliferation and collagen synthesis of a variety of fibroblasts [46,68,69], and Lu et al. proved that PDGF-BB is able to induce phosphorylation of threonine 308 and serine 473 via PI3K, which activates AKT and ultimately triggers tendon cell proliferation and collagen expression, and some of these effects are mediated through the HIF-1a/VEGF signaling pathway [47].

Moreover, PDGF-BB is capable of enhancing α1β1 integrin-mediated cellular collagen matrix remodeling by elevating extracellular signal-regulated kinase (ERK)/activator protein-1 (AP-1) activity. Integrin-mediated signaling has previously been shown to influence cellular behavior together with growth factors, such as migration, proliferation, and survival, among others, while the importance of specific integrin regulation for intra-tendon healing has also been reported [11,70,71]. The ERK/AP-1 signaling pathway, a pathway of great significance for cell migration, plays an active function in PDGF-BB-induced α1β1 integrin-dependent remodeling of the collagen matrix [72]. This conclusion is supported by the following evidence: In the first place, inhibition of ERK1/2 signaling or AP-1 activity significantly reduced α1β1 integrin-induced gel contraction. Next, a remarkable inhibition of PDGF-BB-induced gel contraction and ERK/AP-1 activity was observed in the treatment, functionally blocking anti-α1 Ab and anti-β1 Ab. Finally, α1β1 integrin and PDGF-BB signals appeared to converge on the ERK/AP-1 pathway to enhance the remodeling of the collagen matrix [73].

Additionally, it has been suggested that PDGF-BB does not directly affect collagen synthesis. It acts as a potent chelator of wound macrophages and fibroblasts, possibly by inducing an increase in endogenous TGFβ1, which in turn stimulates new collagen synthesis and enhances wound healing [11,74].

As we all know, mitosis is one of the ways in which an organism’s cells proliferate, and mitotic activity can be used as a measure of the cells’ proliferative potential. PDGF-BB was found to be the most efficacious mitogen of all isoforms of PDGF and enhances mitotic activity in a concentration-dependent manner in the range from 1.0 to 50.0 ng/mL [75]. Both the ras-dependent p42/p44 mitogen-activated protein kinases (MAPKs) and PI3K activated by PDGF-BB are essential for mitogenesis. It is known that the continuous activation of signal transduction pathways, specifically p42/p44 MAPK and PI3K, is necessary for progression through G1 to S [76]. The results of the mitotic assay were very similar to those of the chemotaxis assay; PDGF-BB also showed a strong chemotactic effect, and the maximum effect was observed at 10 ng/mL [77]. It is widely understood that directional decisions during cell migration are crucial for tissue healing as the distribution of cells in the complex pores of the extracellular matrix can determine the outcome of tissue development. In addition to helping cells proliferate, mitosis is one of the influencing factors in the directional decisions of cell migration [78]. Hence, PDGF-BB can be used to influence cell migration through its powerful mitogenic and chemotactic effects, resulting in a more even distribution of cells in the wound, paving the way for tissue remodeling and thus accelerating the tendon healing process [11].

### 4.4. PDGF-BB Induces Cell Differentiation

PDGF-BB acts as a crucial regulator of cellular function as it can promote tendon healing by inducing cell differentiation [79,80]. Src plays a crucial function in many physiological processes, including cell differentiation. PDGF-BB can induce phosphorylation of PDGFRβ and Src in a time-dependent manner, and Src, being an upstream molecule of Janus kinase 2 (JAK2), can promote phosphorylation of JAK2. Finally, PDGF-BB can stimulate cell differentiation by activating the Src/JAK2 signaling pathway [81]. Secondly, protein kinase C-delta (PKC-delta), which is a downstream molecule in the PDGFR signaling pathway, has been shown to possibly have a pivotal effect in PDGFR-β-mediated cell differentiation [82]. It has previously been shown that PDGF can induce the transformation of stem cells into a tendon cell phenotype and thus promote tendon regeneration [83]. Javanshir and Younesi et al. displayed the results of PDGF-BB-induced adipose mesenchymal stem cells’ (ASCs’) differentiation into tendon cells. They observed a significant increase in tendon-specific markers (TNMD and SCX) by qPCR and showed successful tendonization of ASCs by Sirius Red staining, which is a unique method of assessing tendon differentiation. These results proved the feasibility of PDGF-BB as an inducer of ASC tendon cell differentiation [80,84]. Tendonogenic differentiation of ASCs induced by PDGF-BB not only increased the number of tendon-like cells at the injury, but also boosted the mean histological score and the mean collagen fibre tissue score. At the same time, a range of collagen fibre dispersions was shown that was closest to the normal tendon standard [85]. Taken together, PDGF-BB can improve tissue properties by inducing cell differentiation, eventually producing more efficient tendon tissue to aid tendon healing.

## 5. Conclusions and Perspectives

Tendon healing is a complex and lengthy process that involves three overlapping phases. The administration of growth factors is a viable strategy in the event of promoting tendon healing. As a growth factor that accelerates mitogenesis, PDGF-BB has been shown to support tendon healing [13]. PDGF-BB can accelerate the normal tissue repair process by enhancing the repair of early acute inflammatory responses, recruiting and activating relevant immune cells to kill pathogens, and scavenging dead or dying cells [39]. PDGF-BB also induces an angiogenic response during tendon healing, thereby promoting the increase in nutrients and the elimination of metabolic waste products, which further accelerates tendon healing.

Tendon cell proliferation is one of the main responses by adding PDGF-BB in a dose-dependent manner or in complete or serum-free medium [86]. Meanwhile, PDGF-BB has been shown to aid in matrix remodeling by encouraging type I collagen synthesis as well [11]. However, the study by Ojima et al. proposed that the optimal concentration of PDGF-BB for cell proliferation and type I collagen synthesis is different. PDGF-BB stimulates collagen synthesis at low concentrations and inhibits it at high concentrations, which is the opposite of promoting cell proliferation. Such findings provide us with a physiological means: higher concentrations of PDGF-BB are used to promote cell proliferation in the early stages of healing. Subsequently, PDGF-BB at reduced concentrations can be used to enhance the synthesis of type I collagen [87].

Moreover, it has been shown that PDGF-BB can induce the differentiation of ASCs to tendon cells, which facilitates the regeneration of tendon tissue [84]. However, the combination of PDGF-BB and GDF-6 had a more pronounced effect on the induction of the tenogenic differentiation of ASCs [80]. This suggests that the role of a single growth factor is always limited and that synergistic effects of multiple growth factors tend to produce a better result. Additionally, Kim et al. established a rat patellar tendon avulsion model and then found that PDGF-BB and bone morphogenetic protein-2 (BMP-2) could induce tendon differentiation of stem cells after being directly immobilized on heparin-bound polycaprolactone (PCL)/Pluronic F127 membranes, resulting in accelerated regeneration. Based on their findings, they believe that the sustained release of growth factors maximizes their biological function in vivo so that the biological activity of growth factors is maintained long enough for regeneration [88]. In future studies, we should focus on the efficacy of the combination of multiple growth factors in tendinopathies, making full use of the synergistic effects of multiple growth factors so as to stimulate their therapeutic potential and thus better regulate tendon healing.

In recent years, many studies have continuously optimized growth factor delivery strategies, especially PDGF-BB, and developed novel drug delivery devices to control the release of growth factors in time and space to reduce the rapid removal of growth factors at the wound site and then achieve a positive outcome of fostering healing [9,11,15,89]. Moreover, in order to sufficiently exploit the potential of the continuous delivery of growth factors for positive tendon healing, we still need to carefully examine key issues such as the timing of growth factor application, application dose and delivery method. The difference in PDGF-BB administration dose or release rate may also affect the improvement in the structural properties of tendons. For instance, in the study by Thomopoulos et al. a low dose of PDGF-BB (100 ng) was chosen to avoid overstimulation of the administration site and to minimize the risk of adhesion formation between the finger sheath and the tendon. This dose of PDGF-BB caused an increase in bioactivity and enhanced tendon gliding without harmful side effects, but it did not improve tendon repair site strength after three weeks [90]. Although PDGF-BB, a promising candidate for musculoskeletal tissue repair processes, has been demonstrated in many studies to have a role in encouraging tendon healing, the precise mechanisms still need to be elucidated in more detail. Consequently, in future studies, it is essential to further explore the biological response of PDGF-BB in tendon healing in order to better treat them.

## Figures and Tables

**Table 1:** The biological responses embodied by PDGF-BB in different animal experiments.

Study	Animal Type	Models Established	Dosage	Time Post Operation	Outcome	Conclusion
[9]	Rabbits	Achilles tendon of rabbit	PDGF-BB: 8 μg	3 weeks	Tensile strength↑	The tendon tensile strength was increased by a factor of 2 and there was no additional pro-fibrotic effect.
[20]	New Zealand White rabbits	MCL rupture	PDGF-BB: 400 ng, 20 mg	6 weeks	Ultimate load↑ Ultimate elongation values↑	The application of PDGF-BB significantly improved the ultimate load, failure absorption energy and ultimate elongation values of the femoral–MCL–tibial complex. In addition, the high-dose group improved more structural properties of the complex compared to the low-dose PDGF-BB group.
[19]	Horses aged 2–5 years	Superficial digital flexor tendon	rhPDGF-BB: 1, 10, 50, 100 ng/mL	24 h, 48 h, 6 days	Type I collagen↑ Type III collagen↓	The use of rhPDGF-BB may be beneficial for equine tendon repair, particularly through the induction of type I collagen Mrna.
[18]	Dogs	The flexor digitorum profundus tendons.	PDGF-BB: 10 ng/mL	2 h, 24 h	Cell proliferation↑ Collagen synthesis↑	PDGF-BB can stimulate tendon cell proliferation and collagen synthesis.
[8]	Sprague Dawley rats	The supraspinatus tendon	rhPDGF-BB: 30, 100, 300 μg/mL	5 days	Cell proliferation↑ Angiogenesis↑	The rhPDGF-BB enhanced cell proliferation and angiogenesis in the early stages of healing.
[21]	White Leghorn chickens	Flexor tendon repair	PDGF-BB: 10 μg/mL	12 weeks	Cell proliferation↑ Angiogenesis↑	The results showed that the combination of heparinized ELAS with PDGF-BB improved biomechanics and vascularity during tendon healing 12 weeks after primary repair.
[22]	Dogs	Deep toe flexor tendon	PDGF-BB: 5 ng/mL	1, 4 weeks	α5β1↑ αvβ3↑ Cell proliferation↑	PDGF-BB promotes tendon healing by upregulating integrins.
[17]	Swedish Loop rabbits	Deep flexor tendons and extrasynovial peroneal tendons	PDGF-BB: 0.1, 0.3, 1.0, 3.0, 10.0, 30.0, 100.0 ng/mL	4 days	Proteoglycan synthesis rate↑ Collagen synthesis↑ DNA synthesis↑	The effects of PDGF-BB on proteoglycan synthesis rate, collagen synthesis and DNA synthesis were dose-dependent for both medial and proximal synovial internal flexor tendon segments and peroneal extrasynovial tendon segments, and the doses were between 0.1–30 and 0.1–100 ng/mL, 0.1–30 ng/mL; 0.1–30 and 0.1–0.3 ng/mL, between 0.1−30 ng/mL; between 0.1–30 and 0.1–100 ng/mL, between 0.1–10 ng/mL.
[23]	New Zealand White rabbits	MCL rupture and repair	PDGF-BB: 400 ng, 20 mg	1 week	Ultimate load↑ Ultimate elongation↑ Energy of failure absorption↑	PDGF-BB improves the quality of the healing medial collateral ligament, and it may have similar potential to promote healing in other ligaments as well.
[24]	Chicken	Flexor tendons	PDGF-BB: 10, 50, 100 picomolar	8, 24 h	DNA synthesis↑	PDGF-BB stimulates DNA synthesis to promote tendon cell matrix repair.
[25]	New Zealand White rabbits	Deep flexor tendons	PDGF-BB: 10, 50, 100, 150 ng/mL	2, 4, 6 days	Cell proliferation↑ Collagen synthesis↑	Tendons from rabbits showed a significant dose-dependent response to PDGF in the concentration range from 10 ng/mL to 150 ng/mL.
[26]	In vitro	Rat flexor tendon fibroblasts	PDGF-BB: 1, 5, 10, 20, 50, 100, 150, 200, 250 μg/L	12, 24, 36, 48, 60, 72 h	Cell proliferation↑ (Except for 250 μg/L)	PDGF-BB can promote the proliferation of tendon cells in a definite range of concentration and time.
[27]	Rats	MCL rupture and repair	PDGF-BB: 5 μg	2 weeks	Cell proliferation↑ Collagen synthesis↑	PDGF-BB significantly increased the proliferation of fibroblasts and promoted collagen synthesis.
[28]	In vitro	Rabbit ACL and MCL cells	PDGF-BB: 1, 100 ng/mL	12 h	Cell migration↑ (Except for 100 ng/mL)	Low concentrations of PDGF-BB can stimulate cell motility.

↑, significant increase; ↓, significant decrease; ACL, anterior cruciate ligament; MCL, medial collateral ligament; PDGF-BB, platelet-derived growth factor-BB.

## Data Availability

Not applicable.
